# Rationing in the intensive care unit in case of full bed occupancy: a survey among intensive care unit physicians

**DOI:** 10.1186/s12871-016-0190-5

**Published:** 2016-05-03

**Authors:** Anke J. M. Oerlemans, Hub Wollersheim, Nelleke van Sluisveld, Johannes G. van der Hoeven, Wim J. M. Dekkers, Marieke Zegers

**Affiliations:** 1 Radboud University Medical Center, Radboud Institute for Health Sciences, IQ healthcare, P.O. Box 9101, 6500 HB Nijmegen, The Netherlands; 2 Radboud University Medical Center, Department of Intensive Care Medicine, P.O. Box 9101, 6500 HB Nijmegen, The Netherlands

**Keywords:** Critical Care, Ethics, Intensive Care Units, Rationing, Resource Allocation

## Abstract

**Background:**

Internationally, there is no consensus on how to best deal with admission requests in cases of full ICU bed occupancy. Knowledge about the degree of dissension and insight into the reasons for this dissension is lacking. Information about the opinion of ICU physicians can be used to improve decision-making regarding allocation of ICU resources.

The aim of this study was to:

- Assess which factors play a role in the decision-making process regarding the admission of ICU patients;
- Assess the adherence to a Dutch guideline pertaining to rationing of ICU resources;
- Investigate factors influencing the adherence to this guideline.

**Methods:**

In March 2013, an online questionnaire was sent to all ICU physician members (*n =* 761, in 90 hospitals) of the Dutch Society for Intensive Care.

**Results:**

166 physicians (21.8 %) working in 64 different Dutch hospitals (71.1 %) completed the questionnaire. Factors associated with a patient’s physical condition and quality of life were generally considered most important in admission decisions. Scenario-based adherence to the Dutch guideline “Admission request in case of full ICU bed occupancy” was found to be low (adherence rate 50.0 %). There were two main reasons for this poor compliance: unfamiliarity with the guideline and disagreement with the fundamental approach underlying the guideline.

**Conclusions:**

Dutch ICU physicians disagree about how to deal with admission requests in cases of full ICU bed occupancy. The results of this study contribute to the discussion about the fundamental principles regarding admission of ICU patients in case of full bed occupancy.

**Electronic supplementary material:**

The online version of this article (doi:10.1186/s12871-016-0190-5) contains supplementary material, which is available to authorized users.

## Background

The intensive care unit (ICU) is a high pressure environment, where expensive care is delivered by highly qualified personnel to patients suffering from potentially life-threatening conditions. The limited number of ICU beds as well as the pressure of ICU care on the total hospital budget necessitates efficient use of ICU beds and optimal patient flow from ICU to the general ward. Premature discharge is associated with a greater risk of ICU readmissions and with increased morbidity and mortality, and should therefore be avoided if possible [1–3]. Rationing decisions in the ICU, therefore, have to strike a balance between efficient use of resources and preventing premature discharges. Rationing is defined as “allocation of potentially beneficial health care services to some individuals in the face of limited availability that involves the withholding of those services from other individuals” [4].

Decisions to admit a patient are often not merely medical decisions. Non-medical aspects, such as pressure from other healthcare professionals, managers, patients or relatives, may play a role in the decision-making process. Even a clinical judgment such as a patient’s prognosis involves a great deal of subjectivity. Therefore, the ideal of decision-making based on objective medical criteria can be very difficult to achieve. This is especially true when there is an urgent request for an ICU bed from the emergency room, operating theater or general ward while the ICU is (almost) filled to capacity.

Many aspects of intensive care delivery are directed by clinical practice guidelines. Several countries have a guideline pertaining to the situation in which the number of patients that would benefit from ICU care exceeds the number of available ICU beds [5–10]. When comparing these guidelines, two approaches can be distinguished—the first taking a deontological (or duty ethics) approach, the second taking a more utilitarian approach. Concisely put, deontological ethics focuses on the rightness or wrongness of an action itself (by considering whether it is in accordance with a moral rule or duty). Utilitarian ethics, meanwhile, looks at the consequences of an action to judge its morality. The first approach prescribes that in principle, no patients already admitted to the ICU should be transferred to make room for a new admission, since there is a treatment contract between admitted patients and their physician and the hospital, that cannot be terminated unilaterally. That is, unless there is no other option available: if the new admission is highly unstable, is at great risk of further injury or death when transported, and is in need of specific care that cannot be supplied by a nearby hospital, and a patient currently in ICU is stable enough to withstand the risk of transport. The second approach takes a different principle as its basis, and prescribes that the person who stands to benefit most from intensive care, should be admitted to the ICU, or, alternatively, that the person with the lowest transport risk should be the one to be transferred to a different hospital [6–8, 11].

Internationally, there is no consensus on how to best deal with situations of an admission request in cases of full ICU bed occupancy [6, 12–14]. Knowledge about the degree and insight into the reasons for this dissension is lacking. Information about the opinion of ICU physicians can be used to improve decision-making regarding resource allocation in intensive care.

The guideline “Admission request in case of full ICU bed occupancy” of the Dutch Society for Intensive Care (hereinafter to be referred to as “the guideline”) subscribes to the first approach (“no, unless…”) [5]. To provide insight into ICU physicians’ attitudes towards ICU admission decisions and the guideline, this study aimed to:

- Assess which factors play a role in the decision-making process regarding the admission of ICU patients;
- Assess the adherence to a Dutch guideline pertaining to rationing of ICU resources;
- Investigate factors influencing the adherence to this guideline.

## Methods

A questionnaire was developed based on literature and data from individual in-depth interviews and focus group interviews [15–19]. We conducted a total of 25 face to face interviews with ICU physicians and nurses, general ward physicians and nurses (from wards that regularly admit post-ICU patients), ICU managers and post-ICU patients. To explore the themes and dilemmas identified in the individual interviews more in depth, we conducted four focus group interviews with: (1) ICU physicians, (2) ICU nurses, (3) general ward physicians, and (4) general ward nurses. (For a more in depth analysis of these individual and focus group interviews, see Oerlemans et al. [20]).

The questionnaire was pilot tested among two ICU physicians and two independent researchers. The pilot test consisted of completion of the questionnaire and subsequent discussion of the questions together with two of the researchers (AO, NvS).

The online questionnaire was designed using LimeSurvey software, and consisted of 9 demographic questions and 7 questions related to admission requests in case of full bed occupancy (see Additional file 1: Table S1 in the supplemental digital content for details).

In March 2013, an introductory e-mail containing the link to the online questionnaire was sent to all ICU physician members of the Dutch Society for Intensive Care (nearly all Dutch ICU physicians are a member of this society, *n =* 761) working in 90 hospitals, explaining the aim of the study, ensuring the anonymous and confidential handling of data, and inviting them to participate. A reminder was sent two weeks later. Informed consent was implied by completing and sending in the questionnaire.

The questionnaire results were analyzed using Statistical Package for the Social Sciences (SPSS) version 20. Descriptive statistics (counts and percentages) were used for the initial analysis. To enable further statistical analyses, we transformed the continuous variables age, years of experience and number of beds into categorical variables with three categories. To perform further analyses with the scenario-based adherence rate, we converted the results from nominal (four possible answers) to dichotomous (adherence and non-adherence). Chi-square tests and logistic regression analyses were used to compare the respondents’ answers in relation to their demographic variables and hospital characteristics. We regarded *p <* 0.05 as statistically significant. The dataset supporting the conclusions of this article is included within the article’s additional files.

Ethical approval was sought from the Research Ethics Committee of the Radboud University Nijmegen Medical Centre (registration number: 2011/483); the committee judged that ethical approval was not required under Dutch National Law. The anonymity of participants and institutions was maintained in the analysis.

## Results

Of the 761 ICU physician members of the Dutch Society for Intensive Care, 166 physicians (21.8 %) working in 64 different Dutch hospitals (71.1 %) completed the questionnaire (for respondent characteristics, see Table 1). The raw data set with questionnaire results is provided as supplemental digital content (see Additional file 2 in the supplemental digital content for details).

**Table 1:** Respondent characteristics

Respondent characteristics (*n =* 166)	
Gender	
Male (%)	106 (63.9)
Female (%)	57 (34.3)
Missing (%)	3 (1.8)
Age	
≤40 years (%)	60 (36.1)
41–50 years (%)	70 (42.2)
≥51 years (%)	35 (21.1)
Missing (%)	1 (0.6)
Years of experience	
≤5 years (%)	61 (36.7)
6–15 years (%)	72 (43.4)
≥16 years (%)	25 (15.1)
Missing (%)	8 (4.8)
Hospital type	
General (%)	50 (30.1)
Teaching (%)	70 (42.2)
Academic (%)	45 (27.1)
Missing (%)	1 (0.6)
ICU physician training hospital?	
Yes (%)	49 (29.5)
No (%)	112 (67.5)
Missing (%)	5 (3.0)
Number of ICU beds	
0–10 (%)	41 (24.7)
11–25 (%)	71 (42.8)
26+ (%)	53 (31.9)
Missing (%)	1 (0.6)

### Factors influencing ICU admission decisions

Asked how often the number of potential ICU patients exceeds the number of available beds in their ICU, 47.3 % of the respondents indicated this rarely happens, 44.8 % said this happens 1 to 3 times a week, and 7.9 % indicated this happens daily. When asked whether they consider this to be an ethical dilemma, 10.3 % responded “always”, 41.8 % “usually”, 37.0 % “usually not” and 10.9 % “never”. Women were significantly more likely to consider this an ethical dilemma than men (64.9 % vs. 44.8 %, *p =* 0.01).

**Fig. 1:**
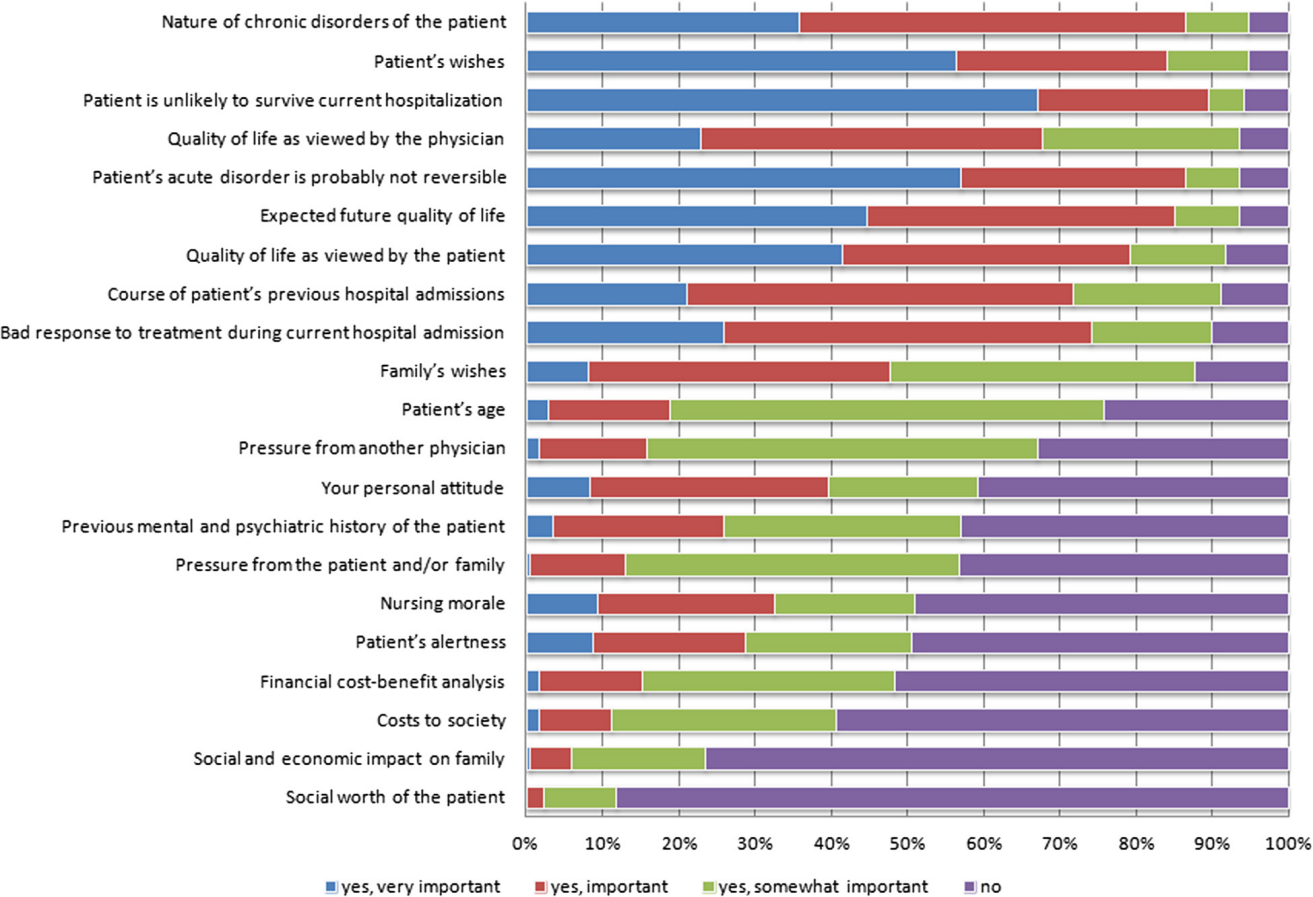
Factors influencing intensive care unit admission decisions

We presented respondents with a list of factors of potential influence in deciding about admission to the last available ICU bed, and asked them to indicate whether they found each factor important (see Fig. 1). The factors associated with a patient’s physical condition (predictors of treatment success) and quality of life were generally considered most important, as were the patient’s wishes. Deemed least important were factors related to the financial burden of treatment for patient, family or society. No significant differences were observed when comparing answers of different genders, years of experience and age of the respondents.

### Guideline adherence

We presented the respondents with a triage scenario (see Table 2), derived from the study of Tallgren et al. (15) Half of our respondents chose the first option (50.0 %), thereby acting according to the guideline. Over one third (40.7 %) chose option two, which is counter to the approach the guideline prescribes, as is option three, which 9.3 % of respondents chose. Those familiar with the guideline were significantly more likely to act in accordance with it than those unfamiliar (*p =* 0.03).

**Table 2:** Responses to triage scenario, derived from Tallgren et al. [15]

You are contacted by the Emergency Room, requesting an ICU bed for an 18-year old male with suspected meningitis and sepsis. The ICU is full. How do you proceed? (*n =* 162)			
	Total (%)	Familiar with guideline (%)	Unfamiliar with guideline (%)
It is my responsibility to take care of the patients who are in the ICU at the moment. Therefore, this is not primarily my concern.	81 (50.0)	40 (59.7)	41 (43.2)
The patient who is likely to benefit the least from care in this ICU is to be treated elsewhere. Therefore, one of the patients in the ICU will be transferred to a regular ward, to a high-dependency unit or to another ICU.	66 (40.7)	19 (28.4)	47 (49.5)
In order to make one more ICU bed available, I will request more nurses to be called to work immediately.	15 (9.3)	8 (11.9)	7 (7.4)
I am not sure what to do. I will consult a colleague for a second opinion.	0 (0)	0 (0)	0 (0)

Scenario-based guideline adherence did not significantly differ between different genders and years of experience. There was a significant association between age category and adherence rate—those aged 51 and over showed lower adherence than those aged between 41 and 50 (*p =* 0.043). Respondents working in an ICU with fewer beds, or working in a general hospital (rather than a teaching or an academic hospital) were significantly more likely to show guideline adherence in their answer to the triage scenario (*p =* 0.013 and *p =* 0.007, respectively).

**Fig. 2:**
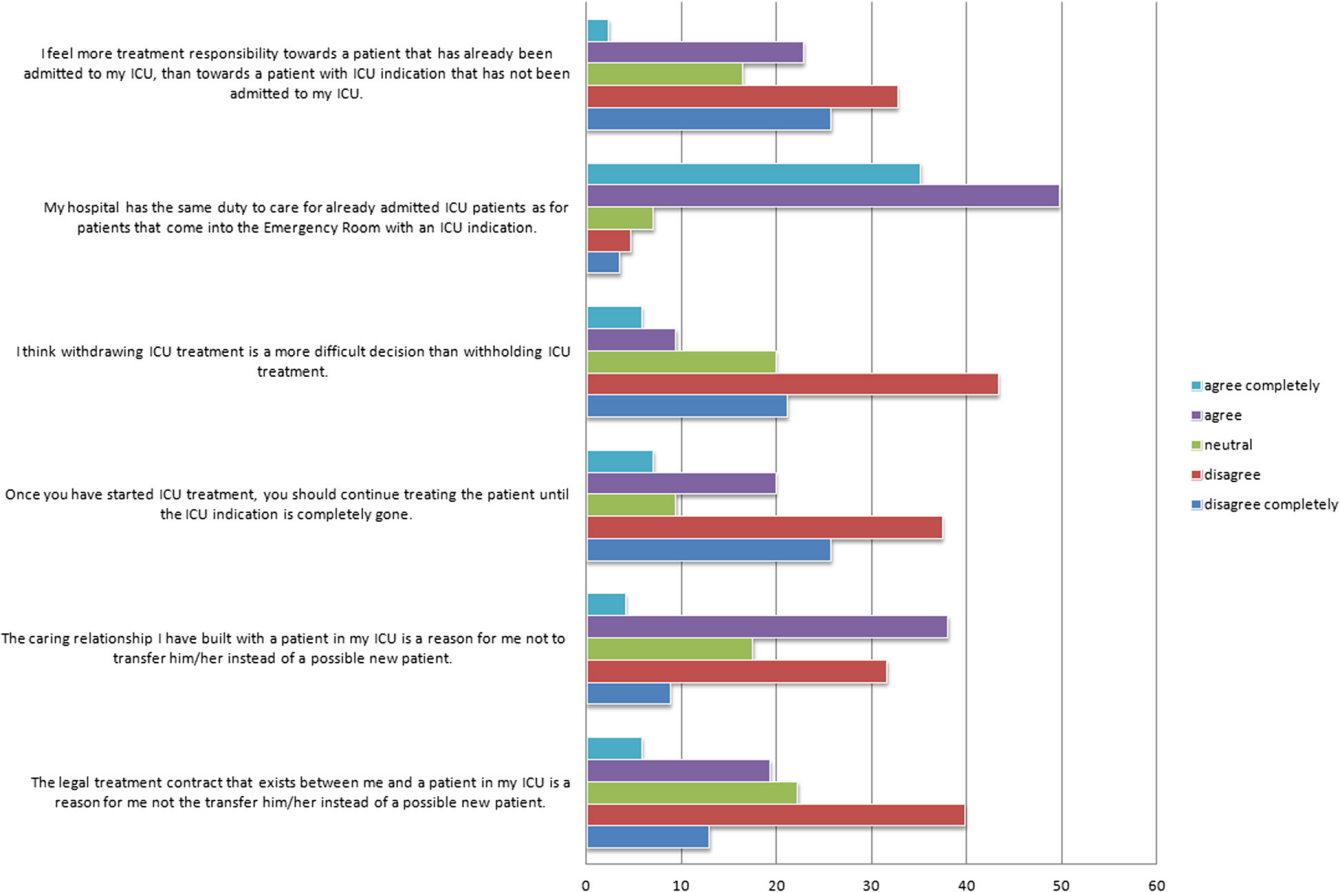
Statements pertaining to admission requests in cases of full intensive care unit bed occupancy

An overwhelming majority of respondents (84.8 %) feel that their hospital has the same duty to care for already admitted ICU patients as for patients with an ICU indication not yet admitted (see Fig. 2). A minority (26.9 %) felt that a patient should always be kept in the ICU until the IC indication is completely gone—meaning that the majority opinion goes against current Dutch guidelines describing admission and discharge criteria.

Respondents who felt more treatment responsibility to a patient currently in their department, or indicated that the caring relationship or the legal contract between them and an admitted patient was a reason not to transfer this patient, tended to answer the triage scenario with the first option (transferring the new patient rather than someone already in ICU). However, this difference was not statistically significant.

### Factors influencing adherence

**Fig. 3:**
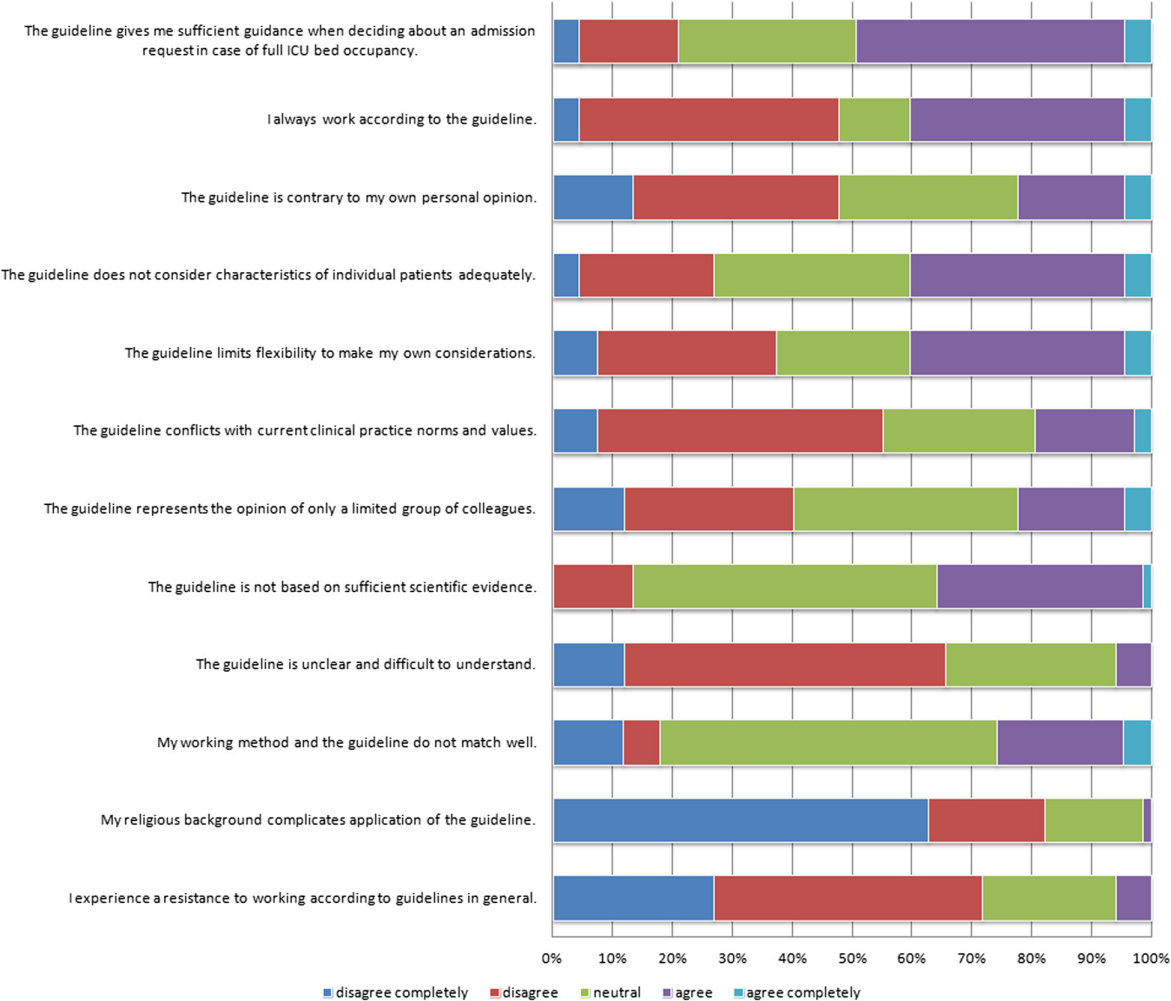
Statements pertaining to the guideline

59.6 % of respondents indicated they were not familiar with the guideline “Admission request in case of full bed occupancy in the ICU”, 40.4 % indicated they were. We asked the 67 respondents familiar with the guideline to respond to a series of statements pertaining to the guideline (see Fig. 3).

For those familiar with the guideline, only about half (47.8 %) indicated that the guideline gives them sufficient guidance in making decisions in cases of full bed occupancy. The majority of respondents indicated that the guideline did not oppose their own opinion, nor did it go against current norms in clinical practice. One-third (35.8 %) felt an adequate scientific basis is lacking, and 40.3 % indicated that the guideline does not sufficiently consider individual patient characteristics. Only 40.3 % indicated to always follow the guideline.

## Discussion

In this study more than half of the respondents (52.7 %) experienced the situation in which the number of patients exceeds the number of available ICU beds at least once a week. When asked whether they considered this to be an ethical dilemma, more than half answered “always” or “usually”. These results imply that situations of full bed occupancy are a regularly occurring, practical as well as an ethical, problem in many ICUs. It is therefore surprising that many respondents were unfamiliar with the guideline that was designed to aid them in these problematic situations. Only half of our respondents indicated that they adhere to the guideline regarding full bed occupancy. Interestingly, where personal characteristics did not seem to be associated with differences in guideline adherence, organizational factors (ICU size and hospital type) did show these significant associations, with respondents from smaller ICUs/hospitals being more likely to adhere to the guideline. This might be due to the fact that smaller hospitals are more used to transferring ICU patients to different (higher level) hospitals, and because they might have fewer opportunities to solve capacity problems in-house by shuttling patients between different departments (which is easier in larger hospitals with more beds, more departments and step-down facilities).

As evidenced by the substantial number of respondents unfamiliar with the guideline (59.6 %) an important explanation for the low adherence may be the (lack of adequate) dissemination of the guideline. Naturally, if people are not familiar with the contents of a guideline, they will not work according to the guideline, at least not consciously. However, a more fundamental problem impacting adherence lies in the disagreement of many ICU physicians with the basic principle underlying the Dutch guideline.

As previously described, two theoretical approaches to triage decisions can be distinguished. The first approach assumes there is an institutional commitment to the patient already in ICU, and an asymmetry of duties of the institution between those receiving intensive care and those not. This assumption is not uncontroversial, however—in their 2012 paper, Hope et al. argued that this may be a fallacy, and institutions in fact have an equal duty of care to all patients of the institution (including those in the emergency room or general wards) [6]. The second approach argues from a more prioritarian perspective: optimizing medical outcomes by prioritizing the sickest patient.

The different approaches are clearly visible when comparing the answers of Dutch and Scandinavian ICU physicians to the triage scenario of Table 2. In Tallgren’s study, no respondents chose to refuse the new patient (compared to 50 % in our study), and 67 % chose to transfer a patient currently in the ICU (compared to 40.7 % in our study). This substantial difference is most likely due to the fact that official recommendations in Scandinavia follow the aforementioned utilitarian approach and thus are counter to the Dutch approach, meaning that option two (i.e. the patient who is likely to benefit the least from care in this ICU is to be treated elsewhere) is the alternative consistent with Scandinavian guidelines [7, 8]. Although many countries have chosen to adopt either the deontological or the utilitarian approach in developing their ICU triage guideline, the right approach to resource allocation in the ICU is still a topic of debate in most of these countries [6, 9–12, 21–27]. The fact that only half of Dutch ICU physicians follow the approach prescribed by the guideline may imply that even if the guideline were adequately disseminated, part of the Dutch ICU physician population may choose to disregard this approach in favor of the one they perceive to be morally just.

While the two approaches may seem disparate, when considering their practical application this may not be the case. In practice, a guideline provides a set of processes or advice, but it is not a mandatory inflexible rule. Therefore, ICU physicians have the option of deviating from the guideline if a particular practice situation requires a different approach. The Dutch guideline, for instance, indicates that in principle no patients already admitted to the ICU should be transferred to make room for a new admission, unless the new admission is at great risk of further injury or death if they were transported and/or required specific care that cannot be supplied by a nearby hospital. Depending on how broadly one interprets this “unless clause”, then, the practical outcome of such a situation can near the result of the second approach.

Using a web-based survey to interview clinicians rather than a paper survey has several advantages with respect to data quality: e.g. higher percentage of questions answered completely and accurately, and lower risk of errors due to manual data entry. Online surveys, however, are associated with lower response rates than paper surveys through regular mail and consequently with the potential for non-response bias [28]. Web-based survey research among healthcare professionals shows substantial variation in terms of reported response rates. Rates below 20 % are not uncommon [28–35]. The response rate to our questionnaire was modest, at 21.8 %. However, when considering the proportion of hospitals with at least one respondent, we managed to include nearly three-quarters of Dutch hospitals. Since we only had access to email addresses, and postal addresses or telephone numbers were not available to us, we were unable to enhance the response rate by using additional methods to reach out to potential respondents. Moreover, for reasons of confidentiality, we could not access demographic data of the non-respondents and were, therefore, unable to analyze the representativeness of our respondents. If respondents do not significantly differ from non-respondents with respect to the relevant characteristics, a low response rate is not necessarily associated with inferior data [28]. Research by Kellerman et al. suggests that the risk of non-response bias may be lower in survey research among physicians than among other populations, possibly since physicians are a relatively homogenous group [36]. In previous studies analyzing non-respondents of survey research, non-response bias was suggested in research in which women, recently licensed physicians and younger physicians were more likely to respond [37, 38]. Our study population consisted of a varied sample in terms of age, experience, and gender.

Ideally, adherence would be assessed by measuring actual behavior. In this case, we did not assess what people do, but rather what they say they do, which could bias our measure of adherence. However, by first asking them to respond to a triage situation, and only afterwards ask respondents directly about the guideline, we hope to have minimized socially desirable behavior.

## Conclusions

In the current economic climate of scarce ICU beds and with an expected future demand for more ICU beds as the population ages and biotechnology enhances survival rates, issues of inequality and injustice in the distribution of critical care services are likely to increase. Adherence to triage guidelines is important, since they provide support regarding the fair and effective use of resources—at least, as long as they were rigorously developed and endorsed by those in the profession. Inadequate dissemination of and disagreement with the fundamental approach underlying the guideline have negatively impacted adherence to the guideline. As Hope et al. describe, in the context of scarcity in the ICU, ethical principles precede clinical guidelines and practical strategies. These ethical principles tell us what we are aiming for in the face of scarce resources, and guidelines then tell us how to achieve what we are aiming for [6]. In addition to scientific evidence about the clinical results of the two triage approaches, discussion about the ethical principles underlying rationing decisions in general and the guideline in particular is a prerequisite for acceptance of the guideline.

### Ethics approval and consent to participate

Ethical approval was sought from the Research Ethics Committee of the Radboud University Nijmegen Medical Centre (registration number: 2011/483); the committee judged that ethical approval was not required under Dutch National Law. Informed consent to participate was implied by completing and sending in the questionnaire.

### Consent for publication

Not applicable.

### Availability of data and materials

The dataset supporting the conclusions of this article is included within the article’s additional files.

### Key messages

- Internationally, there is no consensus on how to best deal with admission requests in cases of full ICU bed occupancy. Information about the opinion of ICU physicians can be used to improve decision-making regarding allocation of ICU resources.
- Scenario-based adherence to the Dutch guideline “Admission request in case of full ICU bed occupancy” was found to be low (50.0 %).
- Unfamiliarity with the guideline and disagreement with the fundamental approach underlying the guideline was associated with this poor compliance.
- In addition to scientific evidence about the clinical results of the two triage approaches, discussion about the ethical principles underlying rationing decisions in general and the guideline in particular is a prerequisite for acceptance of the guideline.

## Additional files

Additional file 1: Table S1. Questionnaire characteristics [15–19]. (DOCX 16 kb)

Additional file 2: Raw data set of questionnaire results. (SAV 40 kb)

## Abbreviations

ICU: intensive care unit
SPSS: statistical package for the social sciences

## References

[1] Chrusch CA, Olafson KP, McMillan PM, Roberts DE, Gray PR (2009). High occupancy increases the risk of early death or readmission after transfer from intensive care. Crit Care Med.

[2] Goldfrad C, Rowan K (2000). Consequences of discharges from intensive care at night. Lancet.

[3] Beck DH, McQuillan P, Smith GB (2002). Waiting for the break of dawn? The effects of discharge time, discharge tiss scores and discharge facility on hospital mortality after intensive care. Intensive Care Med.

[4] Truog RD, Brock DW, Cook DJ, Danis M, Luce JM, Rubenfeld GD, Levy MM (2006). Task Force on Values E: Rationing in the intensive care unit. Crit Care Med.

[5] Commissie ethiek Nederlandse Vereniging voor Intensive Care (NVIC) (2009). Richtlijn in geval van opnamevraag bij volledige bedbezetting op de intensive care.

[6] Hope T, McMillan J, Hill E (2012). Intensive care triage: Priority should be independent of whether patients are already receiving intensive care. Bioethics.

[7] Socialstyrelsen. Livsuppehållande åtgärder i livets slutskede. Stockholm: Socialstyrelsen; 1992.

[8] Ambrosius VM, Huittinen A, Kari H, Leino-Kilpi J, Niinikoski M, Ohtonen V, Rauhala T, Tammisto O (1997). Takkunen: Suomen tehohoitoyhdistyksen eettiset ohjeet [ethical guidelines of the finnish society of intensive care]. Tehohoitolehti.

[9] Guidelines for intensive care unit admission, discharge, and triage (1999). Task force of the american college of critical care medicine, society of critical care medicine. Crit Care Med.

[10] Society of critical care medicine ethics committee (1994). Consensus statement on the triage of critically ill patients. JAMA.

[11] The Intensive Care Society. Guidelines for the transport of the critically ill adult. London: The Intensive CareSociety; 2011.

[12] Osborne M, Patterson J (1996). Ethical allocation of icu resources: A view from the USA. Intensive Care Med.

[13] Skowronski GA (2001). Bed rationing and allocation in the intensive care unit. Curr Opin Crit Care.

[14] Hurst SA, Danis M (2007). A framework for rationing by clinical judgment. Kennedy Inst Ethics J.

[15] Tallgren M, Klepstad P, Petersson J, Skram U, Hynninen M (2005). Ethical issues in intensive care--a survey among scandinavian intensivists. Acta Anaesthesiol Scand.

[16] Einav S, Soudry E, Levin PD, Grunfeld GB, Sprung CL (2004). Intensive care physicians’ attitudes concerning distribution of intensive care resources. A comparison of israeli, north american and european cohorts. Intensive Care Med.

[17] The Society of Critical Care Medicine Ethics Committee (1994). Attitudes of critical care medicine professionals concerning distribution of intensive care resources. Crit Care Med.

[18] Haagen EC, Nelen WL, Hermens RP, Braat DD, Grol RP, Kremer JA (2005). Barriers to physician adherence to a subfertility guideline. Hum Reprod.

[19] Wakkee M, Lugtenberg M, Spuls PI, de Jong EM, Thio HB, Westert GP, Nijsten T (2008). Knowledge, attitudes and use of the guidelines for the treatment of moderate to severe plaque psoriasis among dutch dermatologists. Br J Dermatol.

[20] Oerlemans AJM, van Sluisveld N, van Leeuwen SJ, Wollersheim H, Dekkers WJM, Zegers M (2015). Ethical problems in intensive care unit admission and discharge decisions: a qualitative study among physicians and nurses in the Netherlands. BMC Med Ethics.

[21] Cooper AB, Sibbald R, Scales DC, Rozmovits L, Sinuff T (2013). Scarcity: The context of rationing in an Ontario icu. Crit Care Med.

[22] Moskop JC, Gatter RA (1994). Rationing intensive care. JAMA.

[23] Osborne M, Evans TW (1994). Allocation of resources in intensive care: A transatlantic perspective. Lancet.

[24] Sprung CL, Danis M, Armstrong C, Bailey MA, Dagi TF, Engelhardt HT, Grenvik A, Hofmann P, Hoyt JW, Jameton A, Kofke WA, Lynn J, Marshall MF, Mccartney JJ, Nelson R, Ninos NP, Peduzzi P, Raphaely RC, Rie MA, Rosenbaum SH, Sottille FD, Spanier A, Steinberg A, Tendler MD, Teres D, Truog RD, Wallace T, Yeh TS (1994). Attitudes of critical care medicine professionals concerning distribution of intensive-care resources. Crit Care Med.

[25] Sprung CL, Danis M, Iapichino G, Artigas A, Kesecioglu J, Moreno R, Lippert A, Curtis JR, Meale P, Cohen SL, Levy MM, Truog RD (2013). Triage of intensive care patients: Identifying agreement and controversy. Intensive Care Med.

[26] Sprung CL, Geber D, Eidelman LA, Baras M, Pizov R, Nimrod A, Oppenheim A, Epstein L, Cotev S (1999). Evaluation of triage decisions for intensive care admission. Crit Care Med.

[27] Joynt GM, Gomersall CD, Tan P, Lee A, Cheng CA, Wong EL (2001). Prospective evaluation of patients refused admission to an intensive care unit: Triage, futility and outcome. Intensive Care Med.

[28] Dykema J, Jones NR, Piché T, Stevenson J (2013). Surveying clinicians by web: current issues in design and administration. Eval Health Prof.

[29] Golnik A, Ireland M, Borowsky IW (2009). Medical homes for children with autism: A physician survey. Pediatrics.

[30] Rodriguez HP, von Glahn T, Rogers WH, Chang H, Fanjiang G, Safran DG (2006). Evaluating patients’ experiences with individual physicians: A randomized trial of mail, internet, and interactive voice response telephone administration of surveys. Med Care.

[31] Yusuf TE, Baron TH (2006). Endoscopic transmural drainage of pancreatic pseudocysts: Results of a national and an international survey of ASGE members. Gastrointest Endosc.

[32] VanDenKerkhof EG, Parlow JL, Goldstein DH, Milne B (2004). In Canada, anesthesiologists are less likely to respond to an electronic, compared to a paper questionnaire. Can J Anaesth.

[33] Hofstede SN, van Bodegom-Vos L, Wentink MM, Vleggeert-Lankamp CLA, Vliet Vlieland TPM, de Mheen PJM M-v (2014). Most important factors for the implementation of shared decision-making in sciatica care: ranking among professionals and patients. PLoS One.

[34] Dobrow MJ, Orchard MC, Golden B, Holowaty E, Paszat L, Brown AD, Sullivan T (2008). Response audit of an Internet survey of health care providers and administrators: implications for determination of response rates. J Med Internet Res.

[35] Wiebe ER, Kaczorowski J, MacKay J (2012). Why are response rates in clinician surveys declining?. Can Fam Physician.

[36] Kellerman SE, Herold J (2001). Physician response to surveys: A review of the literature. Am J Prev Med.

[37] Barclay S, Todd C, Finlay I, Grande G, Wyatt P (2002). Not another questionnaire! Maximizing the response rate, predicting non-response and assessing non-response bias in postal questionnaire studies of GPs. Fam Pract.

[38] Cull WL, O’Connor KG, Sharp S, Tang SFS (2005). Response rates and response bias for 50 surveys of pediatricians. Health Serv Res.

