# Supplementary material for: Rationing in the intensive care unit in case of full bed occupancy: a survey among intensive care unit physicians
Source: BMC Anesthesiol. 2016 May 3;16:25. doi: 10.1186/s12871-016-0190-5 (PMC4855768; doi:10.1186/s12871-016-0190-5)
Supplement: Supplementary file 1 — Questionnaire characteristics [15–19]. (DOCX 16 kb) [file 12871_2016_190_MOESM1_ESM.docx]

Table E1. Questionnaire characteristics

| **Question** | **Adapted from** | **Answer modality** |
| --- | --- | --- |
| (1) The frequency of admission requests in case of full ICU bed occupancy | [[15](#_ENREF_15)] | Multiple choice |
| (2) Whether the respondent considers this to be an ethical dilemma | [15] | Multiple choice |
| (3) What factors are deemed important in deciding about admission when there is one available ICU bed | [15-17] | Yes/no with 3-point Likert scale |
| (4) A clinical case about a full ICU | [15] | Multiple choice |
| (5) Statements pertaining to the problem of admission requests in case of full bed occupancy | N/A | 5-point Likert scale |
| (6) Whether the respondent is familiar with the aforementioned guideline | N/A | Multiple choice |
| (7) Statements pertaining to guideline adherence | [18, 19] | 5-point Likert scale |
